# Supplementary material for: Nutrition Literacy Among University Students in Beijing: Status, Determinants, and Implications
Source: Nutrients. 2025 Nov 28;17(23):3748. doi: 10.3390/nu17233748 (PMC12694335; doi:10.3390/nu17233748)
Supplement: Supplementary file 1 [file nutrients-17-03748-s001.zip › Supplement S1 Questionnaire on Nutritional Literacy of College Students in Beijing.pdf]

Questionnaire on Nutritional Literacy of College Students in Beijing

Questionnaire Code:______________________

Interview Date:__________________________

Interview Site:___________________________

Interviewer Name: _______________________

A Letter to Respondent

Dear Participant,

Hello! This is a survey questionnaire on the nutrition literacy status of university students in Beijing conducted by undergraduates from the School of Public Health.Peking University. Before you begin answering the questions.please read the following content carefully and ensure that you fully understand and agree to participate in this survey.

The purpose of this questionnaire survey is to collect information on the nutrition literacy status of university students in Beijing.so that we can better understand the relevant situation and provide references for related research and decision-making.

You will be asked to answer a series of questions related to the research topic. The questionnaire survey is conducted anonymously.and your response data will be kept confidential and will not be disclosed to any third party. We will strictly protect your response data to ensure its security and confidentiality. Your answers will only be used for research purposes and will not be used for commercial purposes. We hope you can answer based on your actual situation. Thank you!

Your participation in this survey is completely voluntary.and you have the right to discontinue or withdraw from the questionnaire survey at any time.

If you have any questions about this questionnaire survey or need further information.please feel free to contact us.

Thank you!

Contact Person: Li Wenpeng

Contact Number: 13391656621

Contact Email: 2210306221@stu.pku.edu.cn

Part 1 Basic Information

1. What is your gender? [Multiple Choice]

A.male B.female

2. What is your specialty? [Multiple Choice]

A. Humanities and Social Sciences Majors B. Science and engineering majors C. Medicine D. Others

3. What is your grade? [Multiple Choice]

A.grade one B. grade two C. grade three D. grade fourand more

4. What is your discretionary monthly living expenses? [Multiple Choice]

A.1,000 yuan or less B.1000-2000 yuan C.2000 yuan - 3000 yuan D.more than 3,000 yuan

5 Have you smoked in the last 30 days? [Multiple Choice]

A.yes B.No

6. What is your usual alcohol consumption? (Please be careful to answer) [Multiple Choice]

A.Always drink alcohol B.Often drink alcohol C.Sometimes drink alcohol D.Rarely drink alcohol

E.Do not drink alcohol at all

7. How do you usually order takeout? [Multiple Choice]

A.Always order takeout B.Often order takeout C.Sometimes order takeout D.Rarely order takeout

E.Never order takeout

8. What is your case with a midnight snack? [Multiple Choice]

A.Always have a midnight snack B.Often have a midnight snack C.Sometimes have a midnight snack

D.Seldom have a midnight snack E.Never have a midnight snack

9. Have you felt any gastrointestinal discomfort in the past 7 days? [Multiple Choice]

A.yes B.No

10. Have you ever been diagnosed with a gastrointestinal disorder? [Multiple Choice]

A.yes B.No

11. How often do you go to the gym? [Multiple Choice]

A. 3 or more times a week B.1-2 times a week C. 1-3 times a month D.Hardly go to it

12. How did you feel about your physical condition in the last week? [Multiple Choice]

A.very good B.better C.general D.poor E.very poor

13. What is your height (cm)? [Fill in the blanks]

____

14. What is your weight (kg)? [Fill in the blanks]

____

15. How has your family been concerned about your diet and giving you advice in the last 30 days? [Multiple Choice]

A.always B.often C.sometimes D.very little E.there is no at all

16. How does your family get nutrition knowledge? [MCQ]

A. Diet and health (health) magazines and books B.short videos (Douyin.station b.etc.). C. Online nutrition articles

D.other ________________ [Fill in the blanks] F.almost no way

17. In which of the following ways have you received dietary and nutrition education? [MCQ]

A. High school organization B. University lectures C. University electives D.community.off-campus activities

E.other ________________ [Fill in the blanks] F.almost no way

18. How often did you eat breakfast in the past week? (Please be careful to answer) [Multiple Choice]

A.every day B.5~6 days C.3~4 days D.1~2 days E.0 days

Part II Nutrition Literacy Evaluation Scale

19. Do you agree with the following statement?

	Strongly agree	Agree	Neutral	Disagree	Strongly disagree
①Rational diet is an important basis for maintaining health and keeping away from diseases.					
②Good dietary patterns are the foundation of adequate nutrition.					
③Food is divided into five categories, including grains and tubers, vegetables and fruits, livestock, poultry, fish, eggs, dairy and other animal food, beans and nuts ,and oils and fats. Different foods have different nutritional characteristics.					
④Whole grains belong to grains and tubers.					
⑤In daily life, we should try not to eat too much and keep a balance between eating and moving.					
⑥A healthy diet should be followed at every stage of life.					
⑦We should cherish food, prepare meals as needed and avoid waste.					
⑧"Separate meals" and " divided meals" are good forms of balanced diet and simple meals.					

⑨We should respect other people's eating customs and pay attention to table manners.					
⑩Nutrition and health food civilization is the core of food civilization.					
⑪Eating with family and friends can boost happiness, as well as appetite.					
⑫The family is the best place to pass on the traditions of good food culture.					

20. Please recall the food you have eaten during the past 7 days, and check the following kinds of food you eat every day on average according to your situation.

For example, if Xiaoming ate steamed bread, rice, kidney beans and sweet potatoes yesterday, he ate four kinds of grains, tubers and miscellaneous beans. He also ate apples, Chinese cabbage and tomatoes, so he ate three kinds of vegetables and fruits：

Foods	Number of kinds of foods		
Grains, tubers and miscellaneous beans	≥ 3	①Yes	②No
Vegetables and fruits	≥ 4	①Yes	②No
Livestock, poultry, fish and eggs	≥ 3	①Yes	②No
Dairy, beans and nuts	≥ 2	①Yes	① No

21. Please recall your life during the past 7 days and answer the following questions according to the actual situation:

During the past 7 days, how many days did you eat the following foods?	7 days	5~6 days	3~4 days	1~2 days	0 day
①Breakfast					
②Coarse grains (potatoes, miscellaneous beans, corn, oats, coarse rice etc.)					
③More than 300g (6 taels) of vegetables					
④Fruits					
⑤Milk, yogurt, powdered milk or cheese (excluding Yo acid milk, Nutri-Express and other dairy drinks)					
⑥Fish, poultry, eggs or lean meat					
⑦Sugary foods (such as bread, cakes, cookies and other snacks)					

⑧Fried, grilled or puffed food					

22. How many glasses of water did you drink per day during the past 7 days? (1 cup =200ml)

①7-8 cups ②5-6 cups ③3-4 cups ④1-2 cups ⑤Not sure.

23. How often do you drink alcohol?

①Always ②Usually ③Sometimes ④Seldom ⑤Never

24. In the past month, how many days per week did you spend more than 30 minutes in total outdoor exercise? (Not including commuting time, shopping time and walking time)

①7 days ②5-6 days ③3-4 days ④1-2 days ⑤0 day

25. What is your attitude to "estimating portion size and matching food is a necessary nutritional skill"?

①Strongly agree ②Agree ③Neutral ④Disagree ⑤Strongly disagree

26. What is the approximate weight of a ping-pong ball-sized egg?

①5g ②50g（1 tael） ③100g（2 taels） ④500g（1 catty） ⑤Not sure

27. The following is the breakfast list of several adults. Which one do you think is the most rational breakfast match?

①Soya milk, whole wheat bread and pickles
②Soya milk, fried dough sticks and pickles
③Milk, fried dough sticks and fruit
④Milk, whole wheat bread and fruit ⑤Not sure

28. Food nutrition label refers to labeling nutrition ingredients and displaying nutrition information on the outer packaging of food, proper nutrition claims and health statements. When you buy packaged food, will you read the food nutrition label?

①Never ②Seldom ③Sometimes ④Usually ⑤Always

29. When you buy food, do you prefer to buy food **without** trans fatty acids?

①Never ②Seldom ③Sometimes ④Usually ⑤Always

30. In the nutritional label, "Nutrient Reference Value%/NRV%" means the percentage of the nutrients in 100 g/ml of the food accounting for daily reference intake. Then, if we only rely on this food to take in protein, how much can we eat to meet our daily needs of protein? (just estimate)

Nutrients	Per 100 g	NRV%
Energy	2012KJ	24%
Protein	6g	10%
Fat	12g	20%

Carbohydrate	62.5g	21%
Sodium	100mg	5%

①400g ②600g ③800g ④1000g ⑤Not sure.

31. What do you value most when you buy qualified raw meat in the supermarket?

①Freshness ②Price ③Packaging ④Brand ⑤Production place

32. Do you or someone who cook at home use grilling and frying?

①Never ②Seldom ③Sometimes ④Usually ⑤Always

33. Do you consider cooking methods when ordering takeout and eating out?

①Never ②Seldom ③Sometimes ④Usually ⑤Always

34. Are you concerned about nutrition information?

①Never ②Seldom ③Sometimes ④Usually ⑤Always

35. What will you do when faced with nutrition knowledge and information from various sources?

①Full acceptance ②Selective acceptance after screening ③No acceptance at all

④Don't have access to nutritional knowledge and information

⑤Not sure.

36. After receiving useful nutritional knowledge and information, do you share it with people around you?

①Never ②Seldom ③Sometimes ④Usually ⑤Always

37. What is your attitude to "health (functional) food can replace medicine"?

①Strongly agree ②Agree ③Neutral ④Disagree ⑤Strongly disagree

38. What is your attitude to "Health (functional) food is a kind of food, which has the common characteristics of general food. And it can adjust the function of human body that is suitable for specific people to eat"?

①Strongly agree ②Agree ③Neutral ④Disagree ⑤Strongly disagree

39. Xiao Ming's recent physical examination found that his bone mineral density index was low, indicating **a high risk of osteoporosis**. Some friends were worried about his health condition and recommended a series of health food to him, such as calcium tablets, multivitamins, fish oil and probiotics. What do you think Xiao Ming should choose?

①Trust your friend's recommendation and buy all at once.

②Do not believe in health food, do not accept the recommendation of friends.

③Compare similar products, and choose the most cost-effective purchase.

④Combine professional advice, product logo, packaging logo, suitable crowd and other factors to choose the one that suits our own comprehensively.

⑤Not sure.

40. What is your attitude to "During the whole process of washing, cutting and storing food, raw and cooked food should be separate"?

①Strongly agree ②Agree ③Neutral ④Disagree ⑤Strongly disagree

41. Do you or someone who cook at home always use different utensils to hold raw and cooked food in daily life?

①Never ②Seldom ③Sometimes ④Usually ⑤Always

42. Before eating edible leftovers from the fridge, what will you do?

① If it does not affect the taste, eat it directly. ②Keep it at room temperature and eat it later.

③Warm it up in a pot or microwave before eating. ④Heat thoroughly (to 100℃ or microwave on high for 2 minutes) before eating.

⑤Not sure.

43. What is your attitude to "Tropical fruits (such as bananas) can be stored in refrigerators"?

①Strongly agree ②Agree ③Neutral ④Disagree ⑤Strongly disagree

44. What is your attitude to "Cooked food should be placed on the top and raw food should be placed on the bottom in refrigerators"?

①Strongly agree ②Agree ③Neutral ④Disagree ⑤Strongly disagree

45. If you fry a plate of braised pork in brown sauce at noon and leave a whole plate, what would you do with it?

①Pour out directly. ②Put it on the table in preservative film or box.

③Put it directly in refrigerator (package without preservative film or box).

⑤Not sure. (do not eat at home or do not handle food)

④Pack it in plastic film or box, and put it in refrigerator.

46. When you order takeout, do you consider the hygiene information of the takeout shop?

①Never ②Seldom ③Sometimes ④Usually ⑤Always

47. When choosing a place to eat out, do you give priority to the sanitary conditions of the dining place?

①Never ②Seldom ③Sometimes ④Usually ⑤Always

48. If you eat out, above which the sanitary level of restaurant will you choose ?

①Level A ②Level B ③Level C ④Pay no attention to

the level.

⑤Not sure.

49. Do you regularly monitor your weight?

①Never ②Seldom ③Sometimes ④Usually ⑤Always

50. Body Mass Index (BMI) index is often used to assess nutritional status of human body, which is defined as weight(kg) / height(m^2). The following table shows the standard range of BMI in China. Xiao Ming weighs 72 kg and is 170 cm tall. Please evaluate his current nutritional status.

**BMI Category**	**Range of BMI**
Underweight	<18.5
Healthy	18.5~23.9
Overweight	24.0~27.9
Obese	≥28.0

①Underweight ②Healthy ③Overweight ④Obese ⑤Not sure.
