# Supplementary material for: Nutrition Literacy Among University Students in Beijing: Status, Determinants, and Implications
Source: Nutrients. 2025 Nov 28;17(23):3748. doi: 10.3390/nu17233748 (PMC12694335; doi:10.3390/nu17233748)
Supplement: Supplementary file 1 [file nutrients-17-03748-s001.zip › Supplementary Tables.pdf]

Supplementary Material

Supplement Table S1 The impact of variables on NL Among College Students(Single factor)

variable		Chi-square value	P-value
Gender		0.69	0.407
	male		
	female		
Major		6.32	0.012
	non-medical		
	medical		
Grade		3.82	0.051
	non-freshman		
	freshman		
School Level		3.96	0.047
	elite		
	standard		
Living expenses (yuan)		11.52	0.003
	0- 2000		
	2000 - 3000		
	3000 and above		
BMI		4.00	0.135
	normal		
	overweight		
	Thin		
Smoke		—	0.713
	no		
	yes		
Drinking alcohol		2.19	0.139
	no		
	yes		
Takeaway frequency		10.43	0.005
	Almost never		
	often		
	Occasionally		
Frequency of midnight snacks		15.03	<0.001
	Almost never		
	often		
	Occasionally		
Gastrointestinal discomfort		0.51	0.477
	no		
	yes		
Gastrointestinal diseases		3.60	0.058
	no		

	yes		
Exercise frequency		19.97	<0.001
	Almost never		
	1-3 times per month		
	1-2 times a week		
	always		
Self -reported health status		19.84	<0.001
	not-good		
	good		
Breakfast frequency		29.72	<0.001
	Almost never		
	3 - 4 days per week		
	5-6 days per week		
	every day		
Family suggestion		3.50	0.174
	Never		
	often		
	Occasionally		
Ways for families to acquire knowledge (types)		6.63	0.010
	less		
	more		
Personal ways of acquiring knowledge (types)		16.87	<0.001
	less		
	more		

*We performed a chi-square test for NL versus other variables, where fisher's exact test was used for to low number of smokers, with each variable defined the same as in the main text.

Supplement Table S2 The relationship between General factors and eating behavior factors

variable	Exercise frequency	Breakfast frequency	Takeaway frequency	Frequency of midnight snacks
gender	<0.001	<0.001	0.099	0.112
major	0.038	0.057	0.403	0.957
grade	0.059	0.026	0.076	0.960
Living expenses (yuan)	<0.001	0.689	<0.001	0.444
Family suggestion	0.006	0.548	0.193	0.852
School Level	0.024	0.003	0.074	0.890
Ways for families to acquire knowledge (types)	0.003	0.028	0.954	0.134
Personal ways of acquiring knowledge (types)	<0.001	0.008	0.315	0.161
BMI	0.166	0.408	0.346	0.452
Drinking alcohol	0.289	0.075	<0.001	0.002
Smoke	<0.001	0.023	0.38	0.076
Gastrointestinal diseases	0.476	0.266	0.033	0.004

Gastrointestinal discomfort	0.148	0.016	0.01	<0.001
Self -reported health status	<0.001	<0.001	0.015	<0.001

*Here, we examined the relationship of each variable between General factors and eating behavior factors using a one-factor rank-sum test.

Supplement Table S3 Correspondence of variable grouping and questionnaire options

variable		Correspondence relationship with the questionnaire items	
		Question number	Option(s)
gender			
	male	1	A. male
	female	1	B. female
major			
	non-medical	2	A. Humanities and Social Sciences Majors；B. Science and engineering majors；D. others
	medical	2	C. medicine
grade			
	non-Freshman	3	B. grade two；C.grade three；D. grade four
	Freshman	3	A. grade one
Living expenses (yuan/month)			
	0- 2000	4	A.1,000 yuan or less; B.1000-2000 yuan
	2000 - 3000	4	C.2000 yuan - 3000 yuan
	3000 and above	4	D.more than 3,000 yuan
Drinking alcohol			
	never	6	E.Do not drink alcohol at all
	yes	6	A.Always drink alcohol；B. Often drink alcohol；C.Sometimes drink alcoho；D.Rarely drink alcohol
Smoke			
	never	5	B.No
	yes	5	A.yes
Takeaway frequency			
	Almost never	7	E.Never order takeout
	Occasionally	7	C.Sometimes order takeout; D.Rarely order takeout
	often	7	A.Always order takeout; B.Often order takeout
Frequency of midnight snacks			
	Almost never	8	E.Never have a midnight snack
	Occasionally	8	C.Sometimes have a midnight snack; D.Seldom have a midnight snack
	often	8	A.Always have a midnight snack; B.Often have a midnight snack
Gastrointestinal diseases			

no	9	B.No
yes	9	A.yes
Gastrointestinal discomfort		
no	10	B.No
yes	10	A.yes
Exercise frequency		
Almost never	11	D.I hardly go to it
Occasionally	11	B.1-2 times a week on average;C. 1-3 times a month on average
often	11	A. 3 or more times a week on average
Self -reported health status		
not-good	12	C.general; D.poor; E.very poor
good	12	A.very good; B.better
Family suggestion		
Never	15	E.there is no at all
Occasionally	15	C.sometimes; D.very little
often	15	A.always; B.often
Breakfast frequency		
Almost never	18	D.1~2 days; E.0 days
3 - 4 days per week	18	C.3~4 days
5-6 days per week	18	B.5~6 days
every day	18	A.every day
School Level		
elite		
standard		
BMI		bmi=height /(weight)^2
normal	13、14	18.5<bmi<24
overweight	13、14	bmi>24
Thin	13、14	bmi<18.5
Ways for families to acquire knowledge (types)		A. Diet and health (health) magazines and books; B.short videos (Douyin.station b.etc.).; C. Online nutrition articles; D.other _________________ [Fill in the blanks] ; F.almost no way
less	16	Choose less than 3 of A-D; or choose F
more	16	Not choose F and choose more than 3 of A-D
Personal ways of acquiring knowledge (types)		A. High school organization; B. University lectures; C. University electives; D.community.off-campus activities; E.other _________________ [Fill in the blanks]; F.almost no way
less	17	Choose less than 3 of A-E; or choose F
more	17	Not choose F and choose more than 3 of

A-E

*Elite colleges can be seen in http://www.moe.gov.cn/s78/A22/A22_ztzl/ztzl_tjsylpt/sylpt_jsgx/201712/t20171206_320667.html

Supplement Table S4 Results of linear regression on factors affecting nutritional literacy

variable	Multivariate linear regression		
	**Score**	**β (95%CI)**	***P***
**Demographic Information**			
Gender (Female: male as Ref.)	67.8±8.57	1.306(-0.006-2.675)	0.061
**Major (Medical: non-medical-Ref.)**	69.9±8.66	0.754(-1.055-2.562)	0.414
Grade (Freshman; non-Freshment as Ref.)	68.2±9.45	0.619(-0.672-1.911)	0.347
**Economic and Physical Data**			
School Level (Elite; Standard as Ref.)	67.9±8.79	0.169(-1.183-1.517)	0.808
Living expenses (CNY/month)			
0- 2000	66.6±9.02	Ref.	Ref.
**2000 - 3000**	**69.0±8.90**	**1.838(0.5437-3.132)**	**0.005**
**3000 and above**	**69.4±9.21**	**2.691(0.568-4.813)**	**0.013**
BMI			
Normal (18.5 ≤ BMI ≤ 24) as Ref	67.8±9.07	Ref.	Ref.
Overweight (BMI > 24)	68.3±9.62	0.861(-0.631-2.353)	0.258
Thin (BMI < 18.5)	66.9±8.18	-0.210(-1.906-1.486)	0.808
**Lifestyle and Health status**			
Drinking alcohol: (Yes; never as Ref.)	67.1±8.90	-1.070(-2.297-0.157)	0.087
Smoke: (Yes; never as Ref.)	67.2±8.86	-0.539(-2.779-3.857)	0.750
**Self -reported health status: (Good; not-good as Ref.)**	**69.9±8.93**	**2.682(1.387-3.977)**	**<0.001**
**Gastrointestinal diseases: (Yes; no as Ref.)**	68.0±9.65	1.031(-0.217-2.280)	0.105
Gastrointestinal discomfort: (Yes; no as Ref.)	66.2±9.17	-0.710(-2.057-0.637)	0.301
**Knowledge Acquisition Paths**			
Family suggestion			
Never as Ref	63.1±8.98	Ref.	Ref.
**Occasionally**	**66.9±9.13**	**2.863(0.448-5.277)**	**0.020**
**Often**	**69.4±8.64**	**4.528(2.081-6.975)**	**<0.001**
Ways for families to acquire knowledge (types): (More; Less as Ref.)	**70.2±9.02**	**1.754(0.339-3.169)**	**0.015**
Personal ways of acquiring knowledge (types): (More; Less as Ref.)	70.2±9.85	1.095(-0.383-2.574)	0.146
**Dietary Behaviors**			
**Exercise frequency**			
Almost never as Ref	66.8±8.72	Ref.	Ref.
**Occasionally**	**70.2±9.61**	**2.368(0.820-3.915)**	**0.003**
Often	71.3±9.00	1.175(-1.560-3.909)	0.400
**Takeaway frequency**			

variable	Multivariate linear regression		
	Score	β (95%CI)	*P*
Almost never as Ref	67.7±12.6	Ref.	Ref.
Occasionally	68.5±9.01	1.598(-1.551-4.747)	0.319
**Often**	65.8±8.35	0.255(-3.092-3.603)	0.881
**Frequency of midnight snacks**			
Almost never as Ref	70.2±9.77	Ref.	Ref.
Occasionally	67.9±8.50	-1.636(-3.270- -0.002)	0.050
**Often**	**63.4±9.58**	**-4.752(-7.066- -2.438)**	**<0.001**
**Breakfast frequency**			
Almost never as Ref	64.6±8.48	Ref.	Ref.
3 - 4 days per week	66.5±9.34	1.331(-0.455-3.117)	0.144
5-6 days per week	**69.2±8.05**	**2.576(0.929-4.224)**	**0.002**
**Every day**	**70.7±9.19**	**3.742(2.123-5.362)**	**<0.001**
